# Preservation of explicit learning of visuomotor sequences during Parkinson’s disease progression

**DOI:** 10.1038/s41598-018-28640-2

**Published:** 2018-07-09

**Authors:** Eriko Kitahara, Yasushi Shimo, Hideo Mori, Masanori Nagaoka

**Affiliations:** 1 Department of Rehabilitation, Juntendo University Hospital, Tokyo, Japan; 2 Department of Neurology, Juntendo University School of Medicine, Tokyo, Japan; 3 Department of Rehabilitation Medicine, Juntendo University Graduate School, Tokyo, Japan

## Abstract

While motor learning approaches are effective in rehabilitating Parkinson’s disease (PD) patients, many studies reported deficits in sequential motor learning in these patients. We hypothesised that preserved explicit learning of visuomotor sequences in PD patients contributed to the effectiveness of motor learning approaches. However, there are very few studies analysing explicit learning of visuomotor sequences during the progression of PD. We investigated this phenomenon in 23 patients with moderate to severe PD (Hoehn–Yahr stages II-IV) and 17 age-matched controls using sequential button-press tasks (2 × 5 task). We found (1) no significant differences in numbers of errors in the 2 × 5 task among control and PD groups. (2) There was a significant difference in response times while exploring correct sequences (ERT) among control and PD groups; ERTs in stage-IV patients tended to be longer than those of control and stage-II groups. (3) All four groups significantly improved their performance (i.e., reduced ERTs in the 2 × 5 task) with sequence repetition, although stage-III:IV patients were slower. Thus, even patients with severe PD can learn visual sequences and can translate them into visuomotor sequences (explicit visuomotor sequence learning), albeit slower than controls, providing evidence for effective motor learning approaches during rehabilitation of patients with advanced PD.

## Introduction

Motor learning approaches and several cognitive strategies were found to facilitate motor sequence learning during the rehabilitation of patients with Parkinson’s disease (PD)^1–7^. Motor learning approaches include processes of action observation and imitation to learn specific actions^6^. Cognitive strategies include the deconstruction of specific behaviours (such as standing up from a chair, navigating an obstacle, or putting on socks) into individual sequential movement components that are learned separately as procedural movements^1^. Both approaches are based on motor sequence learning. However, it is possible that the optimal movement sequence differs between patients with milder vs. advanced stages of PD. If so, this would depend on the level of sequence learning required to accomplish specific behaviours. The planning for effective training of motor sequence learning may require an understanding of how well these processes have been preserved throughout the clinical progression of PD.

Motor sequence learning involves three main processes: (1) cognitive processes for the initial acquisition of explicit visual sequences, (2) associative processes for visuomotor translation from a visual sequence to a motor sequence, and (3) autonomous processes for transfer to automatic movements, which is implicit learning^8–10^. Various methods have been used to examine motor sequence learning processes. The serial reaction time task (SRTT) developed by Nissen & Bullemer^11^ revealed deficits of motor sequence learning in patients with PD by comparing the reaction times between sequentially and randomly ordered series of stimuli with those of age-matched controls^12–16^. Other studies using the SRTT, however, found the opposite results, namely preserved sequence learning in PD patients^17,18^. Seidler *et al*. and Sommer *et al*. reported that patients with PD were able to acquire procedural knowledge of a sequence, but were unable to transfer this knowledge into automatic movements^19,20^. Studies using other tasks to evaluate motor sequence learning demonstrated the deterioration of motor sequence learning in both early^21,22^ and late^23^ phases of learning. Several studies investigated the effects of PD progression on motor learning and suggested that patients with severer motor symptoms showed more impaired motor learning^12,15,16^. Muslimovic *et al*. reported that the patients with more sever motor symptoms, based on Hoehn and Yahr scales, showed a trend towards worse sequence-specific learning^12^. Stephan *et al*. revealed that a higher Hoehn and Yahr score was associated with poor sequence learning^15^. Wilkinson *et al*. reported correlations between the Hoehn and Yahr stage and implicit sequence learning but not for explicit sequence learning^16^. However, visuomotor sequence learning in moderate to severe PD patients has not been well investigated. Especially, there are few reports in stage-IV patients.

**Figure 1:**
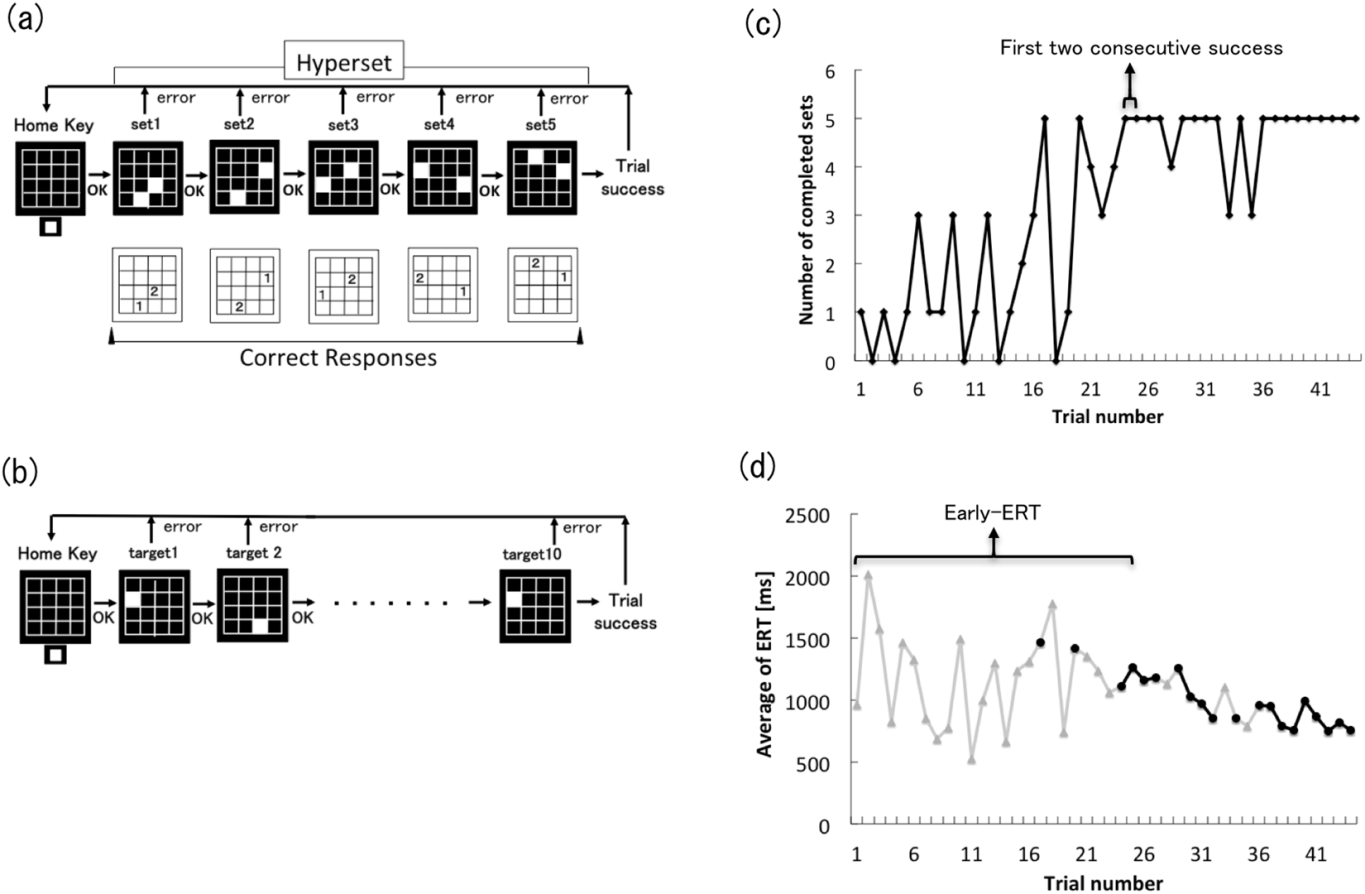
Procedure and outcome measures (**a**) Procedure for the 2 × 5 task. For correct responses, participants were asked to first press the button indicated by a “1” in each set, followed by the one marked “2”. (**b**) Procedure of the random task and 1 × 10 task. (**c**) Number of completed sets in the 2 × 5 task. The performance of a representative patient with stage IV PD. (**d**) The average of ERTs in the 2 × 5 task. These data were obtained by the same patient shown in (**c**). Grey triangles indicate the average ERT in each trial. Black dots indicate the average ERT in successful trials.

The sequential button-press task, also known as the 2 × X task, is a paradigm used to examine motor sequence learning^24^. While the SRTT is considered to involve some perceptual factors^25^, the 2 × X task is thought to demand a greater number of visual sequential memories for the acquisition of sequences. Tremblay *et al*. reported impaired motor chunking during sequence learning in PD patients using a 2 × 7 task^26^. Mochizuki-Kawai *et al*. revealed an inflexibility in PD patients during the learning of new sequences^23^. However, it is still not clear how well cognitive and associative processes in motor sequence learning are preserved in advanced PD patients. We used the 2 × 5 task (Fig. 1a) to assess cognitive and early associative processes in motor sequence learning. We measured the number of errors committed during acquisition of the sequences, the response time (RT) while exploring the sequences, and changes in the RT with sequence repetition. We also used a 1 × 10 task (Fig. 1b) to compare the above parameters with those of associative processes in simple motor sequence learning, which does not require sequence exploration. We further used a random task (Fig. 1b) to assess the visuomotor association between the position of the visual cue and the required response, isolated from sequences. Comparisons of performances in the three tasks among the three PD groups (Hoehn–Yahr stages II-IV) and the control group enabled us to examine the characteristics of visuomotor sequence learning throughout the progression of PD. The aim of this study was to examine to what degree explicit learning of visuomotor sequences is preserved during the progression of PD. We predicted that cognitive processes for the initial acquisition of explicit visual sequences would be preserved and associative process for visuomotor translation would be gradually impaired with PD progression. We hypothesised that preserved explicit learning of visuomotor sequences in PD patients contributed to the effectiveness of motor learning approaches.

## Results

### Subject demographics

The demographics of PD patients and healthy control subjects (controls) are shown in Table 1. There were no significant differences in age, duration of PD, levodopa equivalent daily doses(LEDD), and Mini Mental State Examination (MMSE) scores among the four groups (age; [F(3, 36) = 0.301, P = 0.824], duration of PD; [χ^2^(2) = 3.33, P = 0.19], LEDD; [F(2, 20) = 0.359, P = 0.703], MMSE; [χ^2^(3) = 1.867, P = 0.6]). A significant difference was observed in the motor scores on the Unified Parkinson’s Disease Rating Scale (UPDRS) [F(2, 20) = 11.68, P < 0.001], which were higher in stage-IV patients than in stage-II (P < 0.001) and stage-III (P = 0.014) patients.

**Table 1:** Participant demographics.

Characteristic	PD patients			Controls	F	P	Test used
	Stage II (n = 8)	Stage III (n = 10)	Stage IV (n = 5)	(n = 17)			
Gender (M/F)	5/3	5/5	2/3	10/7			
Age (years)	63.2 ± 5.5	62.8 ± 7.2	61.0 ± 8.5	63.2 ± 5.5	0.301	0.824	ANOVA
MMSE	27.9 ± 1.5	28.3 ± 1.5	28.4 ± 1.5	28.6 ± 1.1		0.6	KW
Duration of PD (years)	7.3 ± 4.5	7.3 ± 3.8	13.8 ± 9.6			0.19	KW
UPDRS motor section	14.4** ± 5.9	22.3* ± 8.9	35.4 ± 7.3		11.7	<0.001	ANOVA
LLED (mg/day)	437.5 ± 240.1	445.0 ± 231.5	550.0 ± 316.2		0.359	0.703	ANOVA

Values are means ± SD. KW: Kruskal Wallis test. *P < 0.05 compared to Stage IV. **P < 0.01 compared to Stage IV.

Subjects were able to use their preferred hands, depending on their hand dominance and symptoms; however, they had to use the same hand in all three tasks. All control subjects used their right, dominant hand for performing the tasks. The 11 patients with unilateral left-side symptoms, the 7 patients with bilateral symptoms, and 3 of the 5 patients with unilateral right-side symptoms all used their right, dominant hand; the other 2 patients with unilateral right-side symptoms were the only ones using the left non-dominant hand.

### 2 × 5 task

#### Number of errors

**Figure 2:**
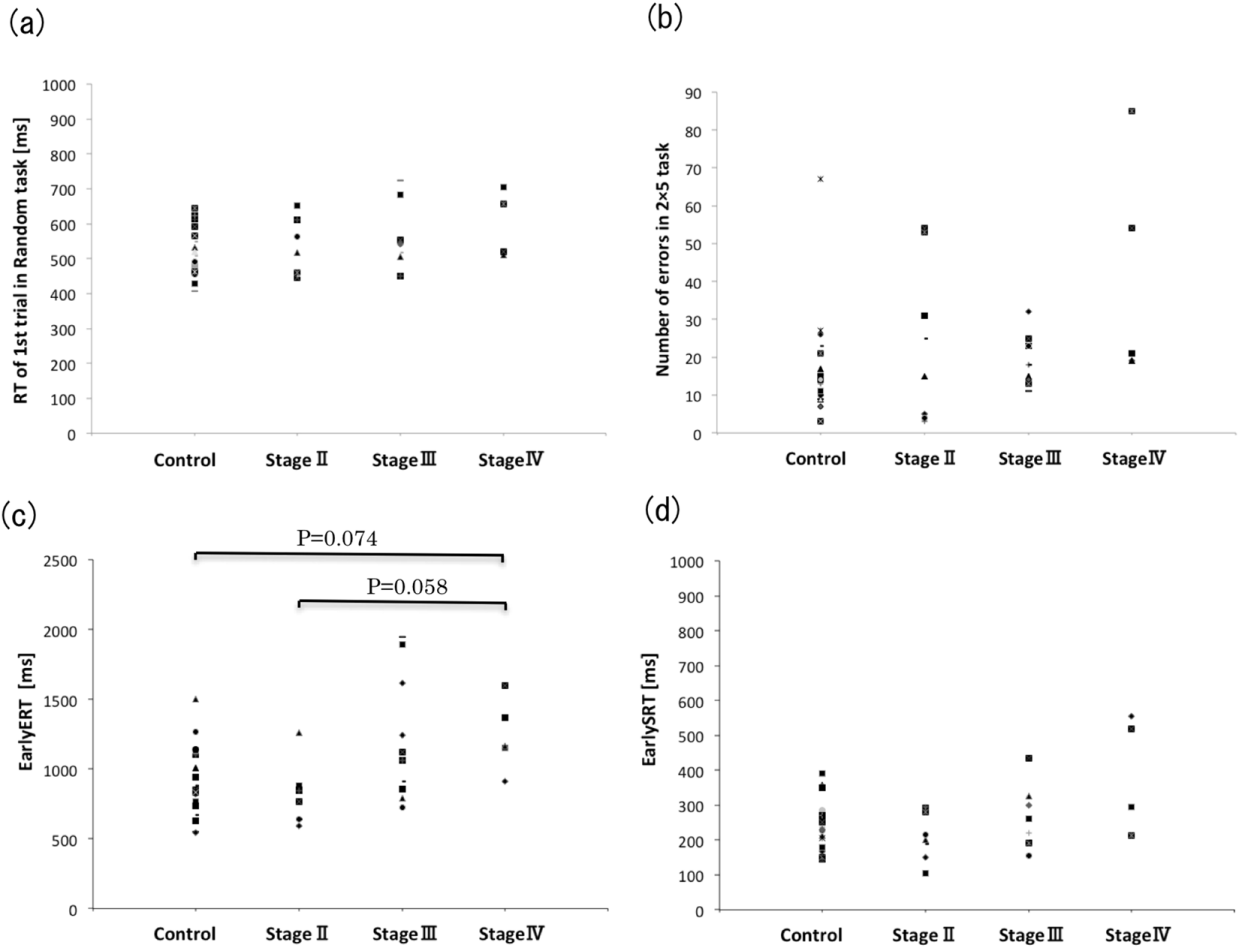
Motor speed in random task and errors and response time in the 2 × 5 task. (**a**) Motor speed in random task. (**b**) The number of errors in the 2 × 5 task for each group. (**c**) Early-ERT in the 2 × 5 task. (**d**) Early-SRT in the 2 × 5 task.

As an indicator of accuracy in the acquisition of the sequence, we recorded the number of errors made before 20 successful trials were completed in the 2 × 5 task (Fig. 1a). As seen in Fig. 1c, a representative patient attained their first success in trial number 17 and their 20th success in trial number 44 after making several mistakes (trials 18, 19, 21, 22, 23, 28, 33, and 35). The numbers of errors committed by each group are shown in Fig. 2b. There were no significant differences in the number of errors made by the control and PD groups (χ^2^(2) = 5.224, P = 0.156).

#### RT for exploring the sequences

We then measured RTs to assess speed of acquisition of the sequences. RTs in the 2 × 5 task were classified into two groups according to the classification system proposed by Watanabe *et al*.^27^: the first RT measure consisted of the time from the illumination of the two target buttons until the first target button was pressed, and the second comprised the time from the release of the first target button until the second button was pressed. We defined the first of these as the exploration RT (ERT) and the second as the simple RT (SRT). We then calculated the average of ERTs before the first two consecutive successful trial completions (for an example, see Fig. 1c) as an indicator of speed in acquiring the visuomotor sequence, and defined this average of ERTs as the Early-ERT (Fig. 1d). There was a significant difference in Early-ERTs among the control and PD groups (χ^2^(3) = 10.2, P = 0.017, Effect size: d = 0.66, Power: 1 − β = 0.92); however, a post hoc comparison did not show significant differences. Although the difference was not statistically significant, Early-ERTs in stage-IV patients tended to be longer than those of the control and stage-II groups, (Steel Dwass test: stage-IV vs. control; P = 0.074, stage-IV vs. stage-II; P = 0.058) (Fig. 2c). We also calculated the average of SRTs before the first two consecutive successful trial, and defined this as the Early-SRT. No significant differences in Early-SRTs were observed among the four groups (χ^2^(3) = 4.72, P = 0.19) (Fig. 2d).

#### Changes in RT with sequence repetition

**Figure 3:**
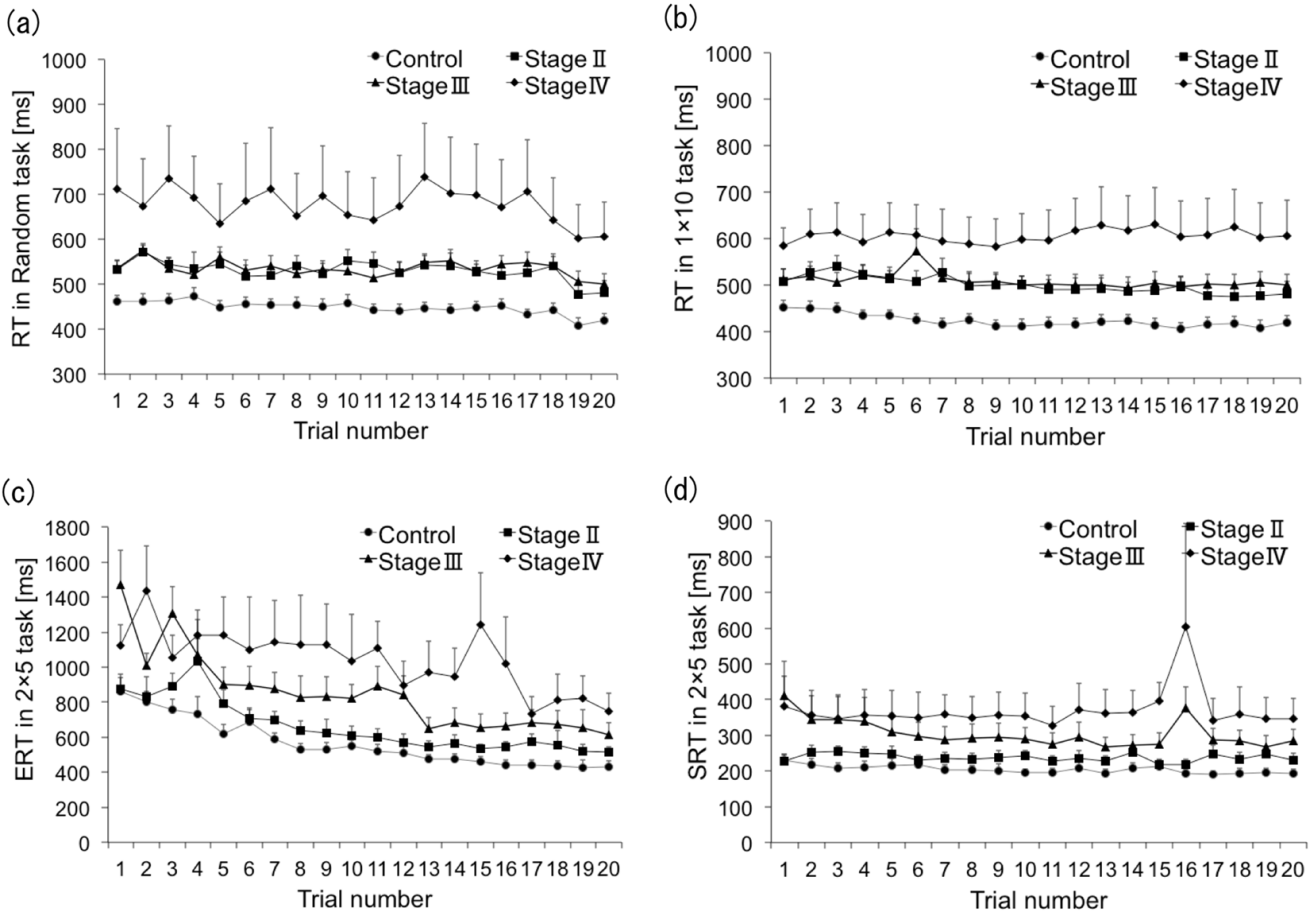
Changes in RT during repeated trials in three tasks. (**a**) RT in the random task. (**b**) RT in the 1 × 10 task. (**c**) ERT in the 2 × 5 task. (**d**) SRT in the 2 × 5 task.

To examine the associative processes for visuomotor translation from a visual sequence to a motor sequence, we assessed changes in the RT with sequence repetition (Fig. 3). For ERT in the 2 × 5 task, a mixed ANOVA indicated that the effect of trial number (F (6.5, 234.1) = 20.9, P < 0.001, d = 0.3, 1 − β = 1.0) and group (F(3, 36) = 5.9, P = 0.002, d = 0.4, 1−β = 1.0) were significant, as was the interaction between trial number and group (F (19.5, 234.1) = 1.9, P = 0.013, d = 0.09, 1 − β = 0.29). During post hoc analyses, we found significant differences between the stage-III and stage-IV groups versus the control and stage-II groups, respectively (stage III vs. control; P = 0.046, stage IV vs. control; P = 0.003, stage IV vs. stage II; P = 0.035). We used regression analyses to examine changes in RT as a reflection of the repetition effect among the control and PD groups. This was carried out for each RT in each task for each participant. The resulting beta values were compared against zero using one-sample *t*-tests at the group level. Analysis of ERT in the 2 × 5 task showed that all four groups significantly improved their performance [control: t(16) = −7.63, p < 0.001 (mean β = −104.08), d = 1.85, 1 − β = 1.0, stage II: t(7) = −5.36, p = 0.001 (mean β = −108.22), d = 1.89, 1 − β = 1.0, stage III: t(9) = −6.14, p < 0.000 (mean β = −155.04), d = 1.94, 1 − β = 1.0, stageIV: t(4) = −3.40, p = 0.027 (mean β = −125.81), d = 1.52, 1 − β = 0.87] (Table 2). We evaluated whether the slopes of these performance curves differed among the 4 groups using a Kruskal Wallis test for beta values; however, there were no significant differences (χ^2^(3) = 2.95, P = 0.4). For the SRT in the 2 × 5 task, a mixed ANOVA indicated that the effect of trial number was not significant (F (2.6, 93.8) = 2.73, P = 0.057), nor was the interaction between trial number and group (F (7.8, 93.8) = 1.5, P = 0.17). In regression analysis, only the control group showed a significant change with repetition of trials [control: t(16) = −2.32, p = 0.034 (mean β = −6.36)].

**Table 2:** Results of one-sample t-test in beta values at the group level.

PD stage	Beta values	Degree of freedom	t values	P
***Random task***				
Control	−22.34 ± 18.12	16	−5.08	<0.001**
Stage II	−8.20 ± 14.21	7	−1.63	0.147
Stage III	−3.33 ± 24.80	9	−0.42	0.681
Stage IV	−3.32 ± 27.56	4	−0.27	0.801
***1*** × ***10 task***				
Control	−18.11 ± 20.51	16	−3.64	0.002**
Stage II	−31.27 ± 22.32	7	−3.96	0.005**
Stage III	−15.18 ± 33.40	9	−1.44	0.185
Stage IV	6.80 ± 35.10	4	0.43	0.687
***2*** × ***5 task ERT***				
Control	−104.08 ± 56.23	16	−7.632	<0.001**
Stage II	−108.22 ± 57.15	7	−5.36	0.001**
Stage III	−155.04 ± 79.8	9	−6.14	<0.001**
Stage IV	−125.81 ± 82.66	4	−3.403	0.027*
***2*** × ***5 task SRT***				
Control	−6.36 ± 11.30	16	−2.32	0.034*
Stage II	−4.26 ± 15.64	7	−0.77	0.47
Stage III	−12.64 ± 44.95	9	−0.89	0.4
Stage IV	11.74 ± 30.04	4	0.87	0.432

Beta values are means ± SD. *P < 0.05 compared to zero, **P < 0.01 compared to zero.

#### Correlational analysis between number of errors as reflected in Early-ERT and beta values

**Figure 4:**
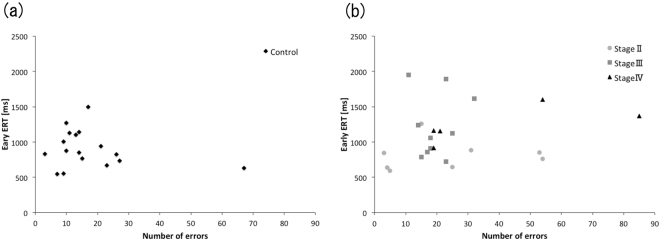
Correlation between number of errors and Early-ERT (**a**) Control group. (**b**) PD group.

To examine characteristics for acquiring visual sequences, we assessed the correlation between the number of errors and Early-ERTs in the control and PD groups. No significant correlations were observed in either group (Spearman test: control: r = −0.098, P = 0.708, PD: r = 0.221, P = 0.312) (Fig. 4). We also assessed the correlation between the numbers of errors and beta values to examine the relationship between cognitive process and associative process. There were also no significant correlations between the numbers of errors and beta values in either group (Spearman test: control: r = −0.13, P = 0.63, PD: r = −0.21, P = 0.34).

#### Correlational analysis between LEDD as reflected in number of errors, Early-ERT, and beta values

To examine the effect of medication on cognitive and associative processes in motor sequence learning, we assessed the correlation between LEDD and the number of errors, Early-ERT, and beta values. No significant correlations were observed (Spearman test: number of errors: r = 0.013, P = 0.952, Early-ERT: r = 0.023, P = 0.917, beta-values: r = 0.124, P = 0.573).

### 1 × 10 task

#### Changes in RT with sequence repetition

We used a 1 × 10 task to assess associative processes in simple motor sequence learning. For RT in the 1 × 10 task, a mixed ANOVA indicated that the effect of trial number was significant (F (5.97, 214.8) = 2.86, P = 0.011, d = 0.12, 1 − β = 0.52), but there was no significant interaction between trial number and group (F (17.9, 214.8) = 1.5, P = 0.082). In regression analysis, the RTs in the control and stage-II groups significantly decreased across sequence repetition [control: t(16) = −3.64, p = 0.002(mean β = −18.11), d = 0.88, 1 − β = 0.97, stage II: t(7) = −3.96, p = 0.005(mean β = −31.27), d = 1.4, 1 − β = 0.97].

### Random task

#### General motor performance

We measured the RT to assess motor speed for pushing the button in the random task, and then calculated the average of 10 RTs in the first trial. No significant differences were observed among the four groups [χ^2^(3) = 1.04, P = 0.79] (Fig. 2a).

#### Visuomotor association

We used the random task to assess the visuomotor association between the position of the visual cue and the required response, isolated from sequences. A mixed ANOVA of RT in this random task indicated that the effect of trial number was significant (F (9.77, 351.7) = 1.87, P = 0.049, d = 0.07, 1 − β = 0.18). The interaction between trial number and group was also significant (F (29.3, 351.7) = 1.73, P = 0.012, d = 0.07, 1 − β = 0.17). Regression analysis showed that only the control group exhibited improved performance across the 20 successful trials [t(16) = −5.08, d = 1.23, 1 − β = 1.0, p < 0.001 (mean β = −22.34)], while none of the three PD groups exhibited significant improvement (Table 2).

## Discussion

This study examined how well explicit learning of visuomotor sequence is preserved during the progression of PD. Our results indicated that (1) cognitive processes for acquisition were largely preserved up to stage IV, (2) associative processes for translating to visuomotor sequences in the 2 × 5 task were preserved up to stage IV; albeit with less improvement in speed in the stage-IV group, and (3) associative processes for translating to motor sequences in the simple sequence task (1 × 10 task) were deteriorated from stage III.

We found no significant differences in the number of errors in the 2 × 5 task among the control and PD groups. A previous study using a 2 × 8 task reported the same result in mild to severe PD patients^19^. Our analyses revealed no significant differences between the stage-IV group and other groups. There was a significant difference in Early-ERT. Stage-IV patients tended to have longer Early-ERTs than the stage-II and control groups. In non-human primates, Miyachi *et al*. reported that the reversible blockade of local neural activity while performing the 2 × 5 task and the blockade of activity in the ventral striatum led to disruptions in the learning of new sequences^28^. In PD patients, degeneration of the substantia nigra innervation to the dorsal striatum is greater than that of the ventral tegmental area innervation to the ventral striatum^29^. Together, our results suggested that the function of the ventral striatum related to learning new sequences is preserved in stage-IV patients. There was also no correlation between numbers of errors and Early-ERT in either the control or PD groups, suggesting that human participants completed the 2 × 5 task using various strategies (for example, short Early-ERT with many errors, long Early-ERT with few errors). Considering these results, we conclude that the cognitive processes to acquire visual sequences were largely preserved up to stage-IV PD.

We observed a significant decrease in ERT with sequence repetition in all groups, suggesting that visuomotor translation is preserved even in patients with advanced PD. Animal experiments using the 2 × 5 task revealed that the pre-sensory motor area coordinates the output of the visual and motor loops, while the premotor cortex controls the translation of visual coordinates into motor coordinates^9,30^. Our results suggest that the coordination and control of the translation between the pre-sensory motor area and the premotor cortex might be preserved in patients with stage-IV PD. However, analysis of the ERTs in this group showed less improvement in speed. Furthermore, only the control group showed a significant decrease in SRT with sequence repetition, suggesting that motor speed improvement with motor repetition was impaired from the early stage of PD. In the 1 × 10 task, the stage-III and stage-IV groups did not exhibit significant decreases in RT with sequence repetition. The SRT and RT in the 1 × 10 task were measured as the response times from perception of visual stimuli to the pushing of the button; in other words, these were determined by perceptual-motor function. On the other hand, in the early phase of the 2 × 5 task, ERTs were mainly determined by working memory to explore correct sequences. We have concluded that reduced ERT and SRT with sequence repetition were the result of working memory and perceptual-motor functions. Our results showed that the clear decline in perceptual-motor function in PD groups might be related to the impaired implicit learning in PD reported by several studies^19,20^. Future studies with increased numbers of trials are needed to confirm whether there is speed improvement (i.e., decreased ERT, SRT, and RT) with more sequence repetition in the 2 × 5 task and the 1 × 10 task.

In interpreting the improvements in speed with repetition, we must consider a measure of the participant’s growing expertise in performing not only the sequence, but also in learning the visuomotor association, or mapping, between the position of the visual cue and the required response^25^. In this study, we examined changes in RT in the random task as an assessment of visuomotor association isolated from sequences. That only the control group showed improved speed with repetition suggests an early deterioration of visuomotor association in PD patients. This suggestion was interesting; however, it should be confirmed in a study with a larger number of trials.

During the rehabilitation of advanced PD patients, practice strategies (e.g., attention, cues, mental rehearsal) for overcoming movement slowness and postural instability when, for example, standing up, turning, and reaching, are recommended^31^. For example, they should be trained with caregivers on what to do if a fall occurs. In a motor learning approach, PD patients observe the demonstration of what to do if a fall occurs and imitate the specific actions with caregivers. A recent systematic review of action observation and motor imagery for rehabilitation in PD patients showed the effectiveness of action observation and less agreement on the effectiveness of motor imagery^32^. Our results on preservation of explicit learning of visuomotor sequences in advanced PD patients provide evidence of the effectiveness of action observation for motor learning approaches in advanced PD patients.

The present study has three important limitations. The first is one of sample size. We examined relatively few stage-II and stage-IV patients, and inclusion criteria of a score equal to or greater than 26 on the MMSE reduced the number of eligible patients. However, our post hoc power analyses of the statistical tests showed that most of them have enough power. Future large-scale studies may provide further important information about the relationships between visuomotor sequence learning function and basal ganglia disorders. Second, we evaluated only 20 successful trials in three tasks. The time required for three tasks, from the start of the explanation to the end of the performance was 20–45 min in the control and PD groups. We also tried to assess multiple hypersets in the 2 × 5 task; however, we had to interrupt them due to fatigue in advanced PD patients. Thus, we may not have observed a sufficient number of trials to properly analyse the process of transitioning from the cognitive to associative stage or that of automatisation of motor sequence learning. As the next step to clarify the number of trials needed for examining translation and automatisation in advanced PD patients, we intend to evaluate RTs in a greater number of successful trials by carrying out the same sequence of tasks over a number of consecutive days. Another noteworthy limitation of our study design is that we evaluated visuomotor sequence learning in PD patients only while they were on their regular medication. Deficits in motor sequence learning have been reported in both ON and OFF dopaminergic medications for PD patients^33–35^. Impairments of early processes for motor sequence learning by dopaminergic medication have been reported in mild to moderate PD patients^36,37^. Furthermore, Kwak *et al*. showed decreased activation of the ventral putamen during PD ON compared to PD OFF^38^. In our study, cognitive and associative processes of the stage-II group were not impaired compared to those of the stage-III and stage-IV groups. Future studies should consider comparisons of the performances of stage-II patients during PD ON and PD OFF periods.

## Conclusion

Our study revealed that even patients with severe PD could learn explicit visual sequences and could translate them into visuomotor sequences, although they showed less improvement in speed. Our findings indicated the potential for motor learning approaches to be effective in advanced PD patients to learn specific actions with caregivers. The optimal strategies in motor learning approaches for advanced PD patients (number of sequences, time for observation of sequences, number of sequence repetition, etc.) need to be investigated in future studies.

## Methods

### Subjects

A total of thirty-seven patients were initially considered for the study. We then excluded patients aged 75 years or older and those affected by dementia, as indicated by a score below 26 on the MMSE. As a result, twenty-three patients with idiopathic PD (12 men, 11 women; mean age 62.9 ± 6.6 years) and 17 age-matched, healthy controls (10 men, 7 women; 63.2 ± 5.5 years) were examined in this study. PD was diagnosed according to the clinical diagnostic criteria of the UK Parkinson’s Disease Society Brain Bank^39^. None of the patients had any history of neurosurgical procedures, stroke, or head trauma. We rated the disease stage using the Hoehn and Yahr disability scale^40^, and classified patients into three groups, stages II, III, and IV. The age-matched healthy control subjects had no neurological abnormalities. The demographics and clinical features of the PD patients and control subjects are shown in Table 1. We assessed the severity of motor symptoms using the motor section of the UPDRS^41^ immediately before the press-button tasks. All patients performed tasks during ON medication. The disease duration was defined as the time interval, in years, between the date of the first PD symptoms, as reported by the patient, and the date of the experiment.

All patients were taking medication: levodopa plus a peripheral levodopa-decarboxylase inhibitor (DCI) (4); levodopa/DCI with a dopamine agonist (4); levodopa/DCI with a dopamine agonist in combination with selegiline, amantadine, entacapone, or an anticholinergic drug (12); levodopa/DCI with entacapone and amantadine (1); dopamine agonist (1); and dopamine agonists paired with anticholinergic drugs (1). In all patients, symptoms were well-controlled by the medication. The LEDD for the three groups are shown in Table 1. This study was approved by the hospital ethics committee, and all participants provided informed written consent. All experiments were performed in accordance with relevant guidelines and regulations.

### Task and procedure

Figure 1a shows the procedure for the 2 × 5 task. At the beginning of each trial, the home key was switched on. When the subject pressed the home key, two of 16 target LEDs turned on simultaneously; this pair was called a “set”. Subjects were required to press the illuminated buttons in the correct order, which was discovered by trial-and-error. If the subject selected the buttons in the correct order, the two buttons turned off as they were pressed, another pair of LEDs was illuminated, and the subject was required again to press these LEDs in the correct order. In total, five sets were presented in a fixed order in each trial; these were called a “hyperset.” The selection of buttons in the correct order was accompanied by a pleasant beep, while an incorrect selection was accompanied by brief illumination of all the LED buttons and an unpleasant beep, ending with abortion of the trial. The subject was then required to start over by pressing the home key to begin a new trial. A trial was designated as successful only when the subject completed the entire hyperset, and the same hyperset was then repeatedly presented. The participants were told that the same hyperset was used in subsequent trials until the subject had successfully completed the set in 20 trials (Fig. 1c). The same hyperset (shown in Fig. 1a) was used for all participants. Before starting the 2 × 5 task, all participants practiced the task procedure using a training hyperset to ensure that they understood the instructions.

Figure 1b shows the procedure used for the 1 × 10 task and the random task. The home key was turned on at the start of the trial, and when pressed by the subject, one of the 16 buttons was illuminated. The subject was required to press the illuminated button, and when pressed, the button turned off and another LED was illuminated. In the 1 × 10 task, ten targets were presented in fixed orders until 20 trials had been successfully performed; RTs were recorded. In the random task, ten targets were presented in a random, non-fixed order. In all three tasks, the subjects were asked to perform the task as quickly and as accurately as possible. After instructions for the tasks and trial practice, participants performed three tasks in the order 1 × 10 task, 2 × 5 task, random task.

### Outcome measures

As the parameter of motor speed for pushing buttons, we measured RT from illumination of the button to pushing the button in both the random and 1 × 10 tasks, and calculated the average of RTs in each trial. In assessing the functioning of cognitive processes, as an indicator of accuracy in the acquisition of a visual sequence, we recorded the number of errors made before 20 successful trials were completed in the 2 × 5 task. We then measured the ERT and SRT to assess the time for acquisition of visual sequences, and calculated the average of ERTs in each trial. While subjects were discovering the correct order of sequences by trial and error, there were both short ERTs (attributed to trying to guess the correct order) and long ERTs (attributed to trying to remembering the correct order). We then calculated the average of ERTs before the first two consecutive successful trial completions (for an example, see Fig. 1c) as an indicator of the time for acquisition of the visuomotor sequence (Early-ERT). After acquisition of the correct order, ERT decreased gradually (Fig. 1d), which we considered to be an aspect of translating visual sequences into visuomotor sequences in associative processing. For associative processes, we assessed changes in ERT and SRT over the course of 20 successful trials, to analyse repetition effects resulting from training. As shown in Fig. 1d, ERT decreased gradually from the first successful trial to the last trial, which we considered to be an aspect of translating visual sequences into visuomotor sequences involved in associative processes.

### Statistical analyses

We used a one-way ANOVA and post hoc comparison (Tukey HSD test) to compare age, scores on the UPDRS part III, and LEDD in the control and PD groups. The Kruskal-Wallis test was used to compare MMSE scores and durations of PD in controls and PD patients. The Kruskal-Wallis test was also used to compare RTs of the 1st trial in the random task among the four groups (PD stages II, III, IV, and control). We compared errors, Early-ERT, and Early-SRT in the 2 × 5 task among the four groups using the Kruskal-Wallis test and post hoc comparison (Steel Dwass test). We used a mixed ANOVA with Trial number (20; successful trials 1–20) as within-subject variable and Group (4; stage II vs. stage III vs. stag IV vs. control) as between-subject variable and post hoc comparison (Tukey HSD test). We also used regression analysis to analyse changes in RT as a reflection of the repetition effect between the control and PD groups. We assessed correlations among number of errors, Early-ERT, and β values using the Spearman test. To compute the achieved power of the statistical tests, we used a post hoc power analysis. Statistical analyses were conducted using the software package SPSS 19.0 (SPSS Inc., Chicago IL, USA), BellCurve for Excel, and G*Power 3.1^42,43^. Statistical significance was defined as P < 0.05 for the main analyses.
